# Proteinuria Detected by Urine Dipstick Test as a Risk Factor for Atrial Fibrillation: A Nationwide Population-Based Study

**DOI:** 10.1038/s41598-017-06579-0

**Published:** 2017-07-24

**Authors:** Woo-Hyun Lim, Eue -Keun Choi, Kyung-Do Han, Tae-Min Rhee, Hyun-Jung Lee, So-Ryoung Lee, Si-Hyuck Kang, Myung-Jin Cha, Seil Oh

**Affiliations:** 1 Division of Cardiology, Department of Internal medicine, Seoul National University Boramae Medical Center, Seoul, Republic of Korea; 2 Division of Cardiology, Department of Internal medicine, Seoul National University Hospital, Seoul, Republic of Korea; 3 Department of Biostatistics, College of Medicine, The Catholic University of Korea, Seoul, Republic of Korea; 4 Department of Cardiology, Cardiovascular Center, Seoul National University Bundang Hospital, Seongnam, Republic of Korea

## Abstract

Proteinuria is one of the well-known risk factors for cardiovascular disease. However the impact of proteinuria on the incidence of atrial fibrillation (AF) is unclear. In this study, we investigated the association between proteinuria detected using urine dipstick test and the risk of AF. A total of 18,201,275 individuals were analyzed, who had no prior AF and had received biennial health checkups provided by the National Health Insurance Service between 2005 and 2008 in Korea. Incidences of AF were ascertained through the end of 2015. During a mean follow-up of 9.6 years, a total of 324,764 (1.8%) developed AF (1.86 per 1,000 person-years). In Cox regression models, proteinuria was associated with an increased risk of AF: adjusted HR and 95% CI of AF occurrence were 1.13 (1.10–1.16), 1.34 (1.31–1.38), 1.53 (1.48–1.58), 1.82 (1.71–1.94), and 1.86 (1.61–2.16) in individuals with trace, 1+, 2+, 3+, and 4+ proteinuria, respectively, compared with those without proteinuria. The result was consistent even after additional adjustment for estimated glomerular filtration rate. In addition, the risk of AF further increased or decreased according to the follow-up dipstick test results. Thus, proteinuria measured with a dipstick test might be considered a potent risk factor for AF development.

## Introduction

Atrial fibrillation (AF) is associated with an increased risk of ischemic stroke and death^1–3^, and it has a significant impact on public health as its prevalence is increasing^4, 5^. Therefore, it is necessary to establish a simple health screening method capable of identifying those with a high risk of developing AF.

Proteinuria is an indicator of renal damage that is often detected earlier than any apparent decline in glomerular filtration rate (GFR). In addition to its role as a risk factor for chronic kidney disease (CKD), proteinuria itself is a predictor for cardiovascular morbidity and mortality, independently of conventional risk factors including CKD^6–9^. However, the impact of proteinuria on the incidence of AF has not been well understood. There are controversial data regarding the association of proteinuria with AF in large cohort studies. The Framingham Heart Study reported that urine albumin-to-creatinine ratio (ACR) was not associated with AF occurrence, whereas the Atherosclerosis Risk in Communities Study demonstrated a relationship between albumin-to-creatinine ratio and the risk of AF^10, 11^.

Urine protein-to-creatinine ratio (PCR) or ACR is commonly used to quantify the level of proteinuria. However, the cost of a PCR or ACR test renders it unsuitable for use in screening for proteinuria in a public health context. On the other hand, the urine dipstick test is a simple and inexpensive diagnostic tool for detecting proteinuria in public health screenings. The urine dipstick test can detect proteinuria with a high sensitivity and specificity of greater than 90%, when using ACR ≥300 mg/g as the reference standard^12, 13^. Dipstick-positive proteinuria is also associated with the risk of death, coronary heart disease, and progression to kidney failure at a given level of estimated GFR (eGFR)^7–9^. In this study, we sought to uncover whether the presence and grade of proteinuria as detected by urine dipstick test could act as a risk factor for AF development in a nationwide population-based cohort.

## Methods

### Data Sources

This study used the whole population database provided by the National Health Insurance Service (NHIS). The NHIS is a mandatory health insurance program managed by the Korean government, which covers 97% of the Korean population (approximately 50 million people). Records from the NHIS database include enrollees’ sociodemographic information, their use of inpatient and outpatient services, and pharmacy dispensing claims. To protect individuals’ privacy, resident registration numbers were encrypted.

Subjects in the NHIS are recommended to undergo standardized health checkups every two years. The health checkups between 2003–2008 included measurements of height, weight, and blood pressure and laboratory tests such as hemoglobin, fasting glucose, cholesterol, liver enzymes, and urinalysis. Data on past medical history, and health-related behaviors such as smoking, alcohol consumption, and physical activity were collected using standardized self-reporting questionnaires. In 2009, creatinine testing was incorporated and the self-reporting questionnaires were modified. This study was exempt from review by the Seoul National University Hospital Institutional Review Board (1607-055-775).

### Study Cohort

We screened Korean residents aged 20 years or older who had undergone at least one of the biennial health checkups provided by the NHIS from January 1, 2005 to December 31, 2008. Individuals with prevalent AF at December 31, 2004 were excluded. A total of 18,201,275 subjects were identified from the NHIS database as the study population, who were followed up to 2015 (Main Cohort). As creatinine levels were measured in the biennial health checkups since 2009, we performed a sensitivity analysis using the Follow-up Cohort to adjust for renal function additionally. The Follow-up Cohort consisted of non-AF subjects who had undergone more than two health checkups at an interval of more than four years. As a result, a total of 15,220,897 subjects were included in the Follow-up Cohort from 2009 to 2013, who were followed up to 2015 (Supplemental Figure).

### Definitions

The primary endpoint of this study was newly diagnosed non-valvular AF during the follow-up period. New-onset non-valvular AF was defined using International Classification of Diseases-Tenth Revision-Clinical Modification (ICD-10-CM) codes (I480–I484 and I489)^14^. Either one diagnosis during hospitalization, or more than two occasions of diagnosis at outpatient clinics were required for the diagnosis of AF. Individuals with a diagnosis of mitral stenosis (I050, I052, and I059) or those with mechanical heart valves (Z952–Z954) were excluded from the analysis. Subjects without AF during the follow-up period were censored at the date of their death or at the end of follow-up, whichever came first.

Baseline comorbidities were also evaluated during the screening period and defined using ICD-10-CM codes, as in our previous study^15–17^. Definitions of covariates are presented in the Supplemental Table.

### Statistical Analysis

Data are presented as mean with standard deviation or numbers with percentages. Incidence rates of AF were calculated by dividing the number of events by 1000 person-years. Cox proportional hazards models were used to evaluate the association between proteinuria on dipstick test and the risk of AF. Model 1 was a Cox proportional model adjusted for age and sex. Model 2 was model 1 with additional adjustment for body mass index, smoking, alcohol consumption, and exercise frequency. Model 3 adjusted for cardiovascular comorbidities such as hypertension, diabetes, and dyslipidemia additionally to model 2. Model 4 was model 3 with additional covariates of ischemic heart disease, congestive heart failure, stroke, and chronic obstructive pulmonary disease. Model 5 was performed only for the Follow-up Cohort, which was model 4 with additional adjustment for estimated GFR. Interactions between variables were tested. Kaplan–Meier curves were plotted to show freedom from AF, and then compared with the log-rank test. Statistical analyses were performed using SAS version 9.2 (SAS Institute, Cary, NC, USA). A two-sided p-value < 0.05 was considered statistically significant.

### Ethical Statement

Ethical approval was waived in this study by the Institutional Review Board (IRB) of Seoul National University Hospital (Seoul, Korea) (#IRB No. E-1607–055–775). Informed consent was not obtained because patient records and information were anonymized and de-identified prior to analysis.

## Results

The baseline characteristics of the study cohort by categories of dipstick proteinuria are summarized in Table 1. The mean age of study population was 45.3 ± 14.6 years and 53.3% were male. Among a total of 18,201,275 study subjects, dipstick urinalysis showed negative protein in 96.76% (n = 17,611,940), trace in 1.49% (n = 270,707), 1+ in 1.18% (n = 214,883), 2+ in 0.46% (n = 83,251), 3+ in 0.10% (n = 17,386), and 4+ in 0.02% (n = 3,108). Subjects with a higher degree of proteinuria were more likely to be older, overweight, with a higher frequency of hypertension, diabetes, dyslipidemia, ischemic heart disease, myocardial infarction, congestive heart failure, stroke, and chronic obstructive lung disease.

**Table 1:** Baseline characteristics of the study population.

Characteristics	Proteinuria by Dipstick test						
	Total	Negative	Trace ( ± )	1+	2+	3+	4+
	(N = 18,201,275)	(N = 17,611,940)	(N = 270,707)	(N = 214,883)	(N = 83,251)	(N = 17,386)	(N = 3,108)
Age	45.3 ± 14.6	45.2 ± 14.6	47.1 ± 14.9	49.5 ± 15.1	51.1 ± 15	52.2 ± 14.7	52.7 ± 14.8
Age group							
<45 years	9,117,474 (50.1)	8,883,857 (50.4)	119,872 (44.3)	80,439 (37.4)	27,321 (32.8)	5,109 (29.4)	876 (28.2)
45–64 years	7,055,518 (38.8)	6,795,612 (38.6)	113,469 (41.9)	97,099 (45.2)	39,287 (47.2)	8,539 (49.1)	1,512 (48.7)
≥65 years	2,028,283 (11.1)	1,932,471 (11.0)	37,366 (13.8)	37,345 (17.4)	16,643 (20.0)	3,738 (21.5)	720 (23.2)
Sex category (Female)	8,508,841 (46.7)	8,228,925 (46.7)	128,619 (47.5)	103,563 (48.2)	38,646 (46.4)	7,739 (44.5)	1,349 (43.4)
Height (cm)	163.5 ± 9.2	163.5 ± 9.2	163.2 ± 9.2	162.5 ± 9.1	162.4 ± 9.1	162.3 ± 9.0	162.3 ± 9.0
Body weight (kg)	63.1 ± 11.4	63.1 ± 11.3	63.8 ± 11.8	64.1 ± 12.1	64.5 ± 12.4	64.6 ± 12.4	64.8 ± 12.3
Body mass index (kg/m^2^)	23.5 ± 3.3	23.5 ± 3.2	23.9 ± 3.4	24.2 ± 3.5	24.4 ± 3.7	24.4 ± 3.7	24.5 ± 3.8
−18.5	788,860 (4.3)	762,830 (4.3)	11,970 (4.4)	9,424 (4.4)	3,735 (4.5)	780 (4.5)	121 (3.9)
18.5–23	7,453,845 (41.1)	7,248,747 (41.2)	100,399 (37.1)	72,093 (33.6)	26,219 (31.5)	5,401 (31.1)	986 (31.7)
23–25	4,409,486 (24.2)	4,272,571 (24.3)	63,800 (23.6)	49,595 (23.1)	18,847 (22.6)	3,964 (22.8)	709 (22.8)
25–30	4,995,496 (27.5)	4,805,162 (27.3)	82868 (30.6)	71,583 (33.3)	28,846 (34.7)	5,989 (34.5)	1,048 (33.7)
30-	553,588 (3.0)	522,630 (3.0)	11,670 (4.3)	12,188 (5.7)	5,604 (6.7)	1,252 (7.2)	244 (7.9)
Systolic blood pressure (mmHg)	123.4 ± 16.6	123.2 ± 16.2	125.2 ± 17.7	128.6 ± 19.1	131.5 ± 20	134 ± 20.6	134.3 ± 21.1
Diastolic blood pressure (mmHg)	77.1 ± 10.9	77 ± 10.7	78 ± 11.5	79.8 ± 12	81.2 ± 12.3	82.1 ± 12.5	81.8 ± 12.6
Smoking status							
Never smoker	11,989,926 (66.0)	11,595,519 (65.8)	179,653 (66.4)	145,118 (67.5)	55,954 (67.2)	11,617 (66.8)	20,65 (66.4)
Ex-smoker	1,488,847 (8.2)	1,437,165 (8.2)	23,468 (8.7)	18,557 (8.6)	7,702 (9.3)	1,647 (9.5)	308 (9.9)
Current smoker	4,722,502 (26.0)	4,579,256 (26.0)	67,586 (25.0)	51,208 (23.8)	19,595 (23.5)	4,122 (23.7)	735 (23.7)
Alcohol drinking							
Complete or near abstinence	9,490,024 (52.1)	9,169,243 (52.1)	142,596 (52.7)	118,856 (55.3)	47,212 (56.7)	10,260 (59.0)	1,857 (59.8)
Moderate consumption							
(≤4 glasses per week)	3,023,297 (16.6)	2,937,260 (16.7)	41,935 (15.5)	30,230 (14.1)	11,291 (13.6)	2,207 (12.7)	374 (12.0)
Heavy drinking							
(≥5 glasses per week)	5,687,954 (31.3)	5,505,437 (31.3)	86,176 (31.8)	65,797 (30.6)	24,748 (29.7)	4,919 (28.3)	877 (28.2)
Exercise							
No	10,009,473 (55.0)	9,688,062 (55.0)	145,150 (53.6)	118,756 (55.3)	46,133 (55.4)	9,647 (55.5)	1,725 (55.5)
1–4 times a week	6,699,138 (36.8)	6,489,208 (36.9)	100,123 (37.0)	74,701 (34.8)	28,229 (33.9)	5,848 (33.6)	1,029 (33.1)
5–7 times a week	1,492,664 (8.2)	1,434,670 (8.2)	25,434 (9.4)	21,426 (10.0)	8,889 (10.7)	1,891 (10.9)	354 (11.4)
Comorbidities							
Hypertension	5,216,608 (28.7)	4,954,357 (28.1)	100,006 (36.9)	101,722 (47.3)	47,273 (56.8)	11,215 (64.5)	2,035 (65.5)
Diabetes	1,636,476 (9.0)	1,513,221 (8.6)	40,662 (15.0)	49,507 (23.0)	25,145 (30.2)	6,633 (38.2)	1,308 (42.1)
Dyslipidemia	2,754,129 (15.1)	2,609,021 (14.8)	55,882 (20.6)	54,806 (25.5)	26,246 (31.5)	6,834 (39.3)	1,340 (43.1)
Ischemic heart disease	457,659 (2.5)	428,605 (2.4)	10,190 (3.8)	11,083 (5.2)	5,903 (7.1)	1,546 (8.9)	332 (10.7)
History of MI	130,590 (0.7)	122,971 (0.7)	2,712 (1.0)	2,864 (1.3)	1,528 (1.8)	427 (2.6)	88 (2.8)
Congestive heart failure	109,719 (0.6)	102,512 (0.6)	2,341 (0.9)	2,772 (1.3)	1,529 (1.8)	457 (2.6)	108 (3.5)
History of stroke	184,509 (1.0)	172,012 (1.0)	4,315 (1.6)	5,014 (2.3)	2,408 (2.9)	633 (3.6)	127 (4.1)
COPD	961,643 (5.3)	922,628 (5.2)	16,087 (5.9)	14,767 (6.9)	6,413 (7.7)	1,454 (8.4)	294 (9.5)
Follow-up (years)	9.6 ± 1.9	9.6 ± 1.9	9.4 ± 2.0	9.2 ± 2.2	9 ± 2.4	8.7 ± 2.7	8.4 ± 2.8

Values are n (%) or mean ± SD.

COPD, chronic obstructive pulmonary disease; MI, myocardial infarction.

During 9.6 ± 1.9 years of follow-up, 324,764 individuals (1.8% of the total population) developed AF. The median time to AF development was 5.3 years. Unadjusted risk factors for AF are presented in Table 2. Both traditional risk factors and proteinuria demonstrated a relationship with AF development, and the degree of proteinuria was shown to be strongly correlated with the risk of AF development.

**Table 2:** Age and sex-adjusted hazard ratios for the risk of atrial fibrillation.

Characteristics	HR (95% CI)	P value
Age		<0.001
<45 years	1	
45–64 years	1.39 (1.37–1.41)	
≥65 years	1.43 (1.39–1.46)	
Female sex	0.65 (0.64–0.65)	<0.001
Height – per 10 cm	1.30 (1.30–1.31)	<0.001
Body weight – per 10 kg	1.28(1.27–1.28)	<0.001
Body mass index		<0.001
Underweight (<18.5 kg/m^2^)	0.98 (0.96–1.00)	
Normal range (18.5–22.9 kg/m^2^)	1	
Overweight (23–24.9 kg/m^2^)	1.13 (1.12–1.14)	
Obese, class I (25–29.9 kg/m^2^)	1.37 (1.36–1.38)	
Obese, class II (≥30 kg/m^2^)	1.93 (1.89–1.96)	
Systolic blood pressure – per 10 mmHg	1.06 (1.06–1.06)	<0.001
Diastolic blood pressure – per 10 mmHg	1.09 (1.09–1.10)	<0.001
Smoking status		<0.001
Never smoker	1	
Ex-smoker	1.07 (1.06–1.08)	
Current smoker	1.01 (1.00–1.02)	
Alcohol drinking		<0.001
Complete or near abstinence	1	
Moderate consumption (≤4 glasses per week)	0.96 (0.95–0.97)	
Heavy drinking (≥5 glasses per week)	1.10 (1.09–1.11)	
Exercise		<0.001
No	1	
1–4 times a week	0.98 (0.98–0.99)	
5–7 times a week	1.03 (1.02–1.04)	
Comorbidities		
Hypertension	1.23 (1.22–1.24)	<0.001
Diabetes mellitus	1.23 (1.21–1.24)	<0.001
Dyslipidemia	0.95 (0.94–0.96)	<0.001
Ischemic heart disease	2.28 (2.26–2.31)	<0.001
History of MI	2.55 (2.48–2.62)	<0.001
Congestive heart failure	3.76 (3.70–3.83)	<0.001
History of stroke	1.61 (1.58–1.63)	<0.001
COPD	1.34 (1.33–1.36)	<0.001
Laboratory findings		
Urine protein		<0.001
Negative	1	
Trace (±)	1.21 (1.18–1.24)	
1+	1.53 (1.50–1.57)	
2+	1.84 (1.78–1.90)	
3+	2.28 (2.13–2.43)	
4+	2.51 (2.17–2.91)	

COPD, chronic obstructive pulmonary disease; CI, confidence intervals; HR, hazard ratio; MI, myocardial infarction

### Proteinuria on dipstick test as an independent risk factor for atrial fibrillation

**Figure 1:**
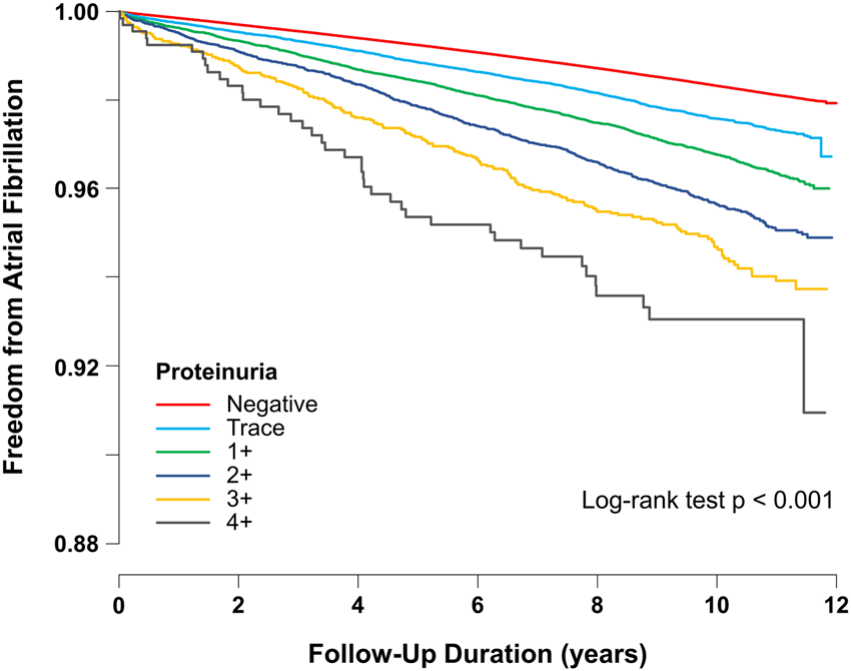
Kaplan-Meier survival curves for freedom from atrial fibrillation. Subjects with higher levels of proteinuria were associated with a higher risk of atrial fibrillation during the follow-up period than those with lower levels of proteinuria.

**Figure 2:**
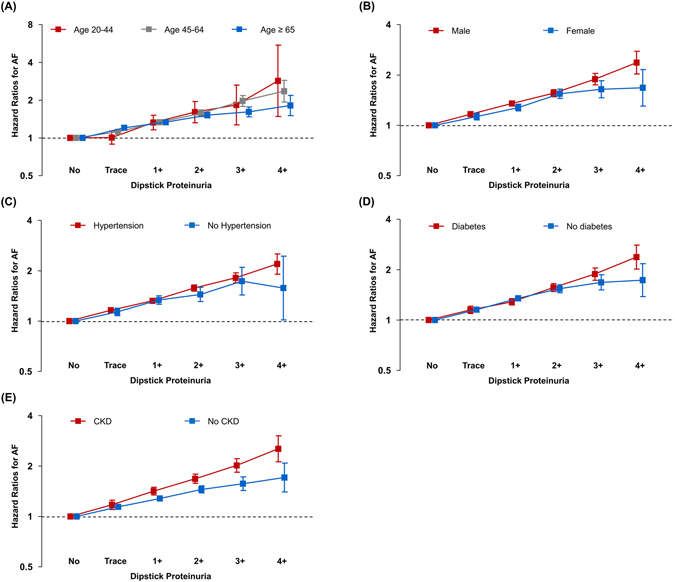
Subgroup analysis. Graded risk of atrial fibrillation development according to the severity of dipstick proteinuria was consistent regardless of (**A**) age, (**B**) sex, (**C**) hypertension, (**D**) diabetes mellitus, and (**E**) chronic kidney disease. Hazard ratios were calculated using a Cox proportional model adjusted for age, sex, body mass index, smoking, alcohol consumption, exercise frequency, diabetes, hypertension, dyslipidemia, ischemic heart disease, congestive heart failure, stroke, and chronic obstructive pulmonary disease, except for the stratification variable. AF, atrial fibrillation; CKD, chronic kidney disease

Figure 1 shows Kaplan-Meier survival curves of freedom from AF for up to 11 years according to the degree of proteinuria detected by dipstick test. The annual incidence rate of AF was 1.8 per 1,000 person-years for the negative proteinuria group, 2.5 for the trace proteinuria group, 3.6 for the 1+, 4.7 for the 2+, 6.1 for the 3+, and 6.8 for the 4+ group, respectively. Higher levels of proteinuria were associated with a higher risk of AF development (p for trend < 0.001). The incidence rate of AF in subjects with proteinuria 4+ was approximately two times higher compared to that in subjects with negative proteinuria (HR 2.05, 95% CI 1.77–2.37, p < 0.001) after adjusting for age, sex, body mass index, smoking, alcohol consumption, exercise frequency, hypertension, diabetes, and dyslipidemia (Table 3, model 3). The HRs were only slightly attenuated after additional adjustment for ischemic heart disease, congestive heart failure, stroke, and chronic obstructive pulmonary disease (HR 1.86, 95% CI 1.61–2.16, p < 0.001, Table 3, model 4). A proportional increase in the risk of AF with the level of proteinuria was consistent regardless of age groups, sex, hypertension, diabetes mellitus, and chronic kidney disease (Fig. 2).

**Table 3:** Incidence and risk of atrial fibrillation according to the degree of proteinuria by dipstick test adjusted for other covariates in the Main Cohort (between 2005 and 2008).

	Proteinuria by Dipstick test						P for trend
	Negative	Trace (±)	1+	2+	3+	4+	
AF cases	306,536	6,379	7,216	3,536	920	177	
Person-years	169,293,090	2,542,006	1,985,107	750,175	151,649	26,213	
AF incidence*	1.81	2.51	3.64	4.71	6.07	6.75	
Model 1 HR (95% CI)	1	1.21 (1.18–1.24)	1.53 (1.50–1.57)	1.84 (1.78–1.90)	2.28 (2.14–2.43)	2.51 (2.17–2.91)	<0.001
Model 2 HR (95% CI)	1	1.19 (1.16–1.22)	1.48 (1.45–1.51)	1.76 (1.70–1.82)	2.19 (2.05–2.33)	2.41 (2.08–2.79)	<0.001
Model 3 HR (95% CI)	1	1.13 (1.11–1.16)	1.36 (1.33–1.39)	1.56 (1.51–1.62)	1.89 (1.77–2.01)	2.05 (1.77–2.37)	<0.001
Model 4 HR (95% CI)	1	1.13 (1.10–1.16)	1.34 (1.31–1.38)	1.53 (1.48–1.58)	1.82 (1.71–1.94)	1.86 (1.61–2.16)	<0.001

AF, atrial fibrillation; CI, confidence intervals; HR, hazard ratios

*Per 1000 person-years

Model 1: Cox proportional model adjusted for age and sex. Model 2: model 1 with additional adjustment for body mass index, smoking, alcohol consumption, and exercise frequency. Model 3: model 2 with additional adjustment for diabetes, hypertension, dyslipidemia. Model 4: model 3 with additional adjustment for ischemic heart disease, congestive heart failure, stroke, and chronic obstructive pulmonary disease.

### Proteinuria on dipstick and graded risk of AF after adjustment of kidney function

**Figure 3:**
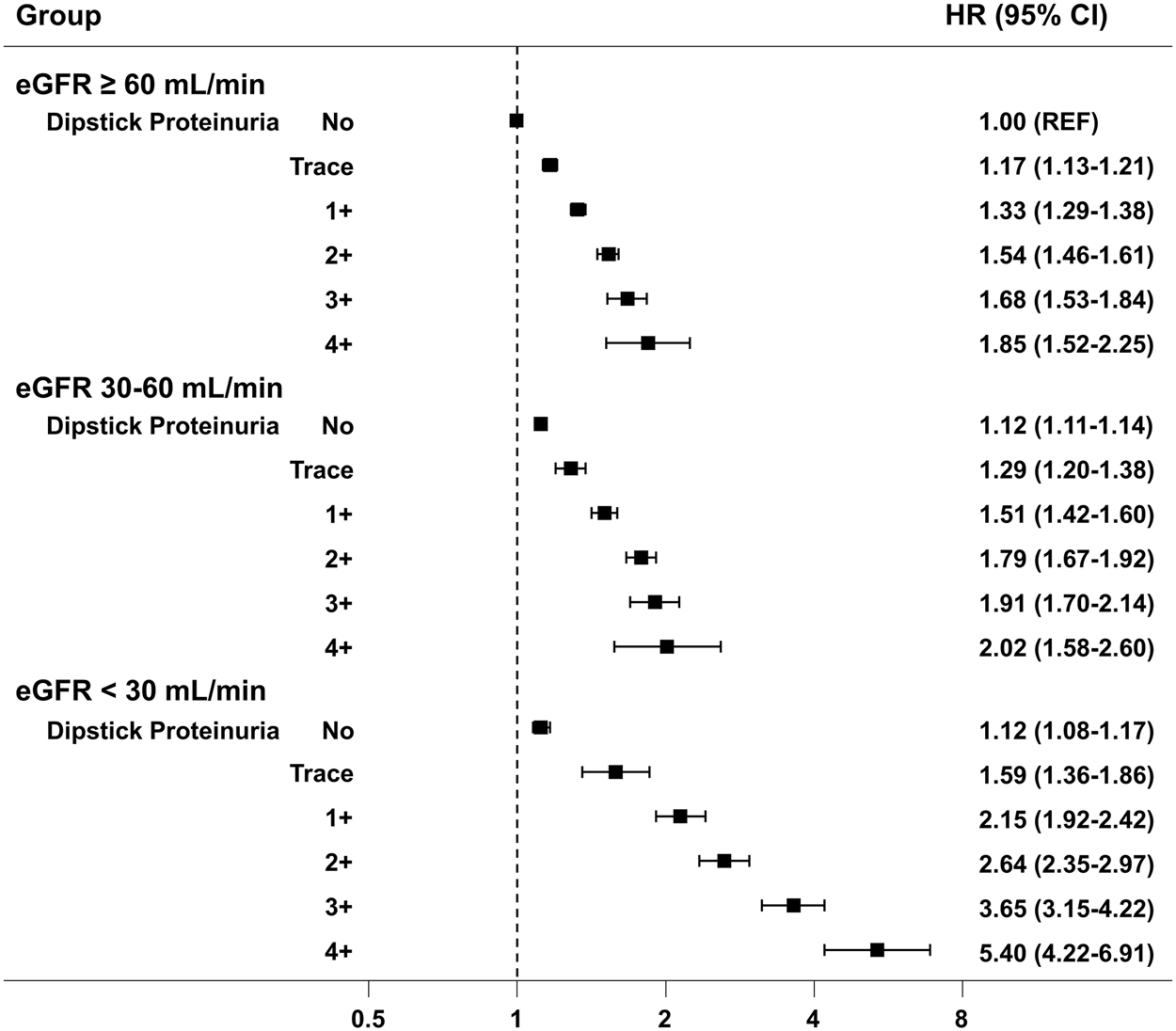
The risk of atrial fibrillation (AF) according to the dipstick proteinuria and estimated glomerular filtration rate in the Follow-up Cohort. The positive correlation between atrial fibrillation development and the degree of dipstick proteinuria was consistent across all three categories of estimated glomerular filtration rate. Hazard ratios were calculated using a Cox proportional model adjusted for age, sex, body mass index, smoking, alcohol consumption, exercise frequency, diabetes, hypertension, dyslipidemia, ischemic heart disease, congestive heart failure, stroke, and chronic obstructive pulmonary disease. AF, atrial fibrillation; eGFR, estimated glomerular filtration rate

To adjust the impact of kidney function on the risk of AF, a sensitivity analysis was performed using the Follow-up Cohort which included data on serum creatinine levels. eGFR was calculated using the Cockcroft-Gault equation and added to the multivariable analysis. After adjustment for potential confounders including eGFR, there was a graded risk of AF according to the severity of dipstick proteinuria (p for trend < 0.001, Table 4, model 5). The positive correlation between AF risk and the degree of dipstick proteinuria was consistent across all three categories of eGFR (<30 mL/min, 30~60 mL/min, and ≥ 60 mL/min,). The association between the grade of proteinuria and AF risk was strongest in the group with eGFR < 30 mL/min compared with other groups (Fig. 3).

**Table 4:** Incidence and risk of atrial fibrillation according to the degree of proteinuria by dipstick test adjusted for other covariates in the Follow-up Cohort (between 2009 and 2013).

	Proteinuria by Dipstick test						P for trend
	Negative	Trace (±)	1+	2+	3+	4+	
AF cases	153,673	5,045	5,160	2,691	920	227	
Person-years	79,960,610	1,878,771	1,331,291	503,385	129,868	25,398	
AF incidence*	1.92	2.69	3.88	5.35	7.08	8.94	
Model 1 HR (95% CI)	1	1.24 (1.21–1.28)	1.51 (1.47–1.55)	1.88 (1.80–1.95)	2.32 (2.18–2.48)	2.74 (2.40–3.13)	<0.001
Model 2 HR (95% CI)	1	1.22 (1.19–1.26)	1.46 (1.42–1.50)	1.80 (1.73–1.87)	2.22 (2.08–2.37)	2.65 (2.32–3.03)	<0.001
Model 3 HR (95% CI)	1	1.16 (1.12–1.19)	1.34 (1.30–1.38)	1.58 (1.52–1.64)	1.88 (1.76–2.01)	2.24 (1.96–2.56)	<0.001
Model 4 HR (95% CI)	1	1.15 (1.12–1.19)	1.32 (1.29–1.36)	1.55 (1.49–1.62)	1.81 (1.69–1.93)	2.13 (1.86–2.43)	<0.001
Model 5 HR (95% CI)	1	1.15 (1.11–1.18)	1.30 (1.27–1.34)	1.51 (1.45–1.57)	1.73 (1.62–1.85)	2.03 (1.77–2.31)	<0.001

AF, atrial fibrillation; CI, confidence intervals; HR, hazard ratios.

*Per 1000 person-years.

Model 1 to 4: Cox proportional models as in Table 3. Model 5: model 4 with additional adjustment for estimated glomerular filtration rate.

### Change in proteinuria on dipstick and risk of AF

We sought to determine whether there is difference in the risk of AF according to the change in the dipstick test results over time, using the Follow-up Cohort. Among individuals who had undergone more than two dipstick tests, two test results were selected that were at least four years apart. Most subjects (n = 14,126,994, 92.8%) who showed initially negative dipstick results were still negative on subsequent tests. However, in those with trace or positive results during the follow-up period, the risk of AF increased significantly after adjustment for covariates (Table 5). Among those whose proteinuria progressively increased, the risk of AF increased by nearly 40% (negative to≥1+, HR 1.36, 95% CI 1.33–1.39; trace to ≥1+, HR 1.38, 95% CI 1.25–1.52). In contrast, in subjects whose proteinuria was resolved, the risk of AF decreased significantly. In subjects who showed trace results at baseline and a negative result on the follow-up test, there was no significant change in the risk of AF compared to those whose levels remained trace on the follow-up test (HR 1.06, 95% CI 1.02–1.10; HR 1.09, 95% CI 0.94–1.27). However, in those with greater than 1+ levels of proteinuria at baseline and a negative result on the follow-up test, the risk of AF decreased compared to those who remained at the same level (HR 1.15, 95% CI 1.10–1.19 vs. HR 1.69, 95% CI 1.61–1.77).

**Table 5:** Risk of atrial fibrillation according to the follow-up dipstick test.

Change in proteinuria			Subject number	AF cases	HR (95% CI)				
					Model 1	Model 2	Model 3	Model 4	Model 5*
Negative	→	Negative	14,126,994	148,173	1	1	1	1	1
Negative	→	Trace	320,945	4,534	1.23 (1.19–1.26)	1.21 (1.17–1.24)	1.15 (1.11–1.18)	1.14 (1.11–1.18)	1.14 (1.10–1.17)
Negative	→	≥1+	304,660	6,753	1.58 (1.55–1.62)	1.53 (1.50–1.57)	1.40 (1.36–1.43)	1.38 (1.34–1.41)	1.36 (1.33–1.39)
Trace	→	Negative	194,523	2,470	1.13 (1.09–1.18)	1.11 (1.07–1.16)	1.07 (1.02–1.11)	1.06 (1.02–1.11)	1.06 (1.02–1.10)
Trace	→	Trace	11,555	179	1.23 (1.06–1.43)	1.19 (1.03,1.38)	1.10 (0.95–1.27)	1.11 (0.96–1.29)	1.09 (0.94–1.27)
Trace	→	≥1+	15,033	406	1.74 (1.58–1.92)	1.65 (1.50–1.82)	1.44 (1.31–1.59)	1.42 (1.29–1.57)	1.38 (1.25–1.52)
≥1+	→	Negative	182,234	3,030	1.33 (1.28–1.37)	1.28 (1.24–1.33)	1.18 (1.14–1.22)	1.16 (1.12–1.20)	1.15 (1.10–1.19)
≥1+	→	Trace	13,641	332	1.73 (1.55–1.93)	1.66 (1.49–1.85)	1.46 (1.31–1.63)	1.42 (1.27–1.58)	1.38 (1.24–1.54)
≥1+	→	≥1+	51,312	1,839	2.31 (2.21–2.42)	2.19 (2.09–2.30)	1.84 (1.75–1.93)	1.78 (1.70–1.86)	1.69 (1.61–1.77)

AF, atrial fibrillation; CI, confidence intervals; HR, hazard ratios.

Model 1 to 5: Cox proportional models as in Table 4. Covariates adjusted in these models were acquired at the time of the secondary dipstick urine tests.

*In model 5, estimated glomerular filtration rate was calculated based on the follow-up health checkup data.

## Discussion

To the best of our knowledge, this is the largest population-based study to examine the impact of the proteinuria dipstick test on the risk of AF. The present study demonstrated the following findings: (1) Proteinuria detected using a dipstick test was significantly associated with an increased risk of AF development; (2) there was a graded risk of AF according to the severity of dipstick proteinuria; (3) dipstick proteinuria was an independent risk factor for AF, even after accounting for potential confounders, including GFR; and (4) the risk of AF increased or decreased according to the results of the follow-up dipstick test.

Proteinuria, particularly microalbuminuria, can be observed in the absence of any evident renal disease and earlier than any apparent decline in GFR, even in the nondiabetic and non-hypertensive population^18^. In such cases, urinary protein excretion reflects not only subclinical renal disease, but also generalized endothelial dysfunction^19, 20^. Moreover, proteinuria is more frequent in patients with diabetes mellitus, high blood pressure, and diseases that cause chronic inflammation, all of which are established risk factors for AF^21–23^. In addition, several studies recently reported that AF is closely associated with the presence of proteinuria^11, 24–26^. Therefore, proteinuria may indeed serve as a sentinel marker for AF risk.

It is generally recommended to measure ACR or PCR in “spot” urine samples to quantify and qualify the degree of proteinuria^27^. The urine dipstick test has a high sensitivity and specificity in screening proteinuria when compared with PCR or ACR. An Australian cohort study reported that a dipstick result of at least 1+ or greater identified ACR≥300 mg/g with 98.9% sensitivity and 92.6% specificity^12^. In the Korean elderly population, a cutoff value of 1+ also exhibited a high sensitivity and specificity (95.6% and 92.2%, respectively) in detecting ACR ≥300 mg/g and PCR 0.5 g/g (sensitivity, 95.6%; specificity, 86.9%)^13^. Therefore, dipstick proteinuria of 1+ or greater can reliably identify significant proteinuria. Although a dipstick test could detect proteinuria with a high sensitivity and specificity, it could generate unacceptable false-positive rates in population-based screening since the prevalence of significant proteinuria is low in the general population^28^. Furthermore, the grading of proteinuria is only semi-quantitative and dependent on urine concentration. A dipstick result of 1+ refers to approximately 30 mg of protein per dL; 2+ refers to 100 mg/dL; 3+ to 300 mg/dL, and 4+ to 1,000 mg/dL^29^. Nonetheless, the urine dipstick test is still recommended as an initial test for the evaluation of CKD^27^ and is widely used, particularly in population-based health screening, due to its simplicity and low-cost^30, 31^. In this study, proteinuria on dipstick test, conducted in a context of population-based screening, was proven to be a strong risk factor for AF development.

CKD is a well-known risk factor for cardiovascular disease^32^, reduced kidney function is associated with increased risk of not only coronary heart disease, heart failure, and mortality, but also AF^11, 33, 34^. Lower eGFR was reported to be associated with incident AF even within the normal or mildly impaired range^35^. Since proteinuria is a primary marker of kidney damage and is associated with poorer outcomes in CKD patients, it is difficult to elucidate the impact of proteinuria on the development of AF. In our study, after adjusting for kidney function either by inclusion of eGFR as a covariate or by subgroup analysis according to the presence of CKD, we found that proteinuria is in fact an independent risk factor for AF development.

Proteinuria itself is also a risk factor for cardiovascular outcomes, independently of conventional risk factors including CKD^6–9^. The Reduction in Endpoints in Non–insulin dependent diabetes mellitus with the Angiotensin II Antagonist Losartan (RENAAL) study showed the renal protective effect of losartan in patients with diabetic nephropathy, which resulted in the reduction of proteinuria and an improvement in cardiovascular outcomes^36^. Although we do not have a definite explanation regarding how proteinuria has decreased in our study, we found that the decrease in proteinuria between serial dipstick tests was associated with the reduction in AF risk in the general population. Interestingly, in individuals whose proteinuria had progressed from negative to ≥1+, the risk of AF increased by approximately 40%. Proteinuria on dipstick test was shown to be an independent risk factor for AF development. However, it was also found to be a modifiable risk factor for AF development.

Our study has several limitations. First, this study is a nationwide population-based retrospective observational study, which is susceptible to several biases including selection bias. The NHIS provides biennial health checkups to all health insurance subscribers, but only half of the subjects actually received these medical examinations. Therefore, the study population most likely included those who maintain healthier lifestyles, or those who are more concerned about their health. Second, the incidence of AF was based entirely on claim data and there could have been undetected or unreported incidences of AF. There is an inherent possibility of underestimation of silent AF because population studies using claim data do not, by nature, screen subjects directly with ECG. Conversely, misclassification of AF diagnostic codes could lead to overestimation of AF. Frequent and long-term monitoring is reported to improve the detection rate of AF^37, 38^. Third, a baseline urine dipstick protein was obtained by a single measurement. This may lead to random measurement error and regression dilution bias^39, 40^, which tends to underestimate the real association between proteinuria and AF development. Since dipstick urinalysis is a semi-quantitative test, it has its own variability. Changes in the level of proteinuria during the follow-up may also dilute the impact of proteinuria on the risk of AF development. Although we did not correct the regression dilution bias directly, we assessed the risk of AF development according to the change in dipstick proteinuria over time, which showed that the risk of AF development increased by approximately 40% among those whose proteinuria grade increased. Fourth, in the current study, data regarding prescription medication use were not available. Studies have shown that angiotensin-converting enzyme inhibitors (ACEIs) and angiotensin II receptor blockers (ARBs) reduce proteinuria and protect against deterioration in renal function in patients with diabetic nephropathy^41^. ACEIs and ARBs are also known to prevent new-onset AF^42^. Thus, data on prescription medications, especially ACEIs and ARBs, would have enriched our results. Fifth, this study was composed of an entirely Korean population. Therefore, the results may not be generalizable to other ethnicities. Lastly, the self-reported questionnaires might have a limited quality of information and lack verification.

In conclusion, we found that proteinuria detected by urine dipstick test is a potent risk factor for AF development. There was graded risk of AF according to the severity of dipstick proteinuria. Moreover, the risk of AF was shown to decrease or increase according to the results of follow-up dipstick tests. The urine dipstick test could be a simple and easy way to predict the risk of AF in the general population. However, further investigations are needed to determine whether dipstick test screening could be used as a method to stratify individuals at risk of AF who require more intensive monitoring and stroke prevention.

## Electronic supplementary material

Supplemental Table and Figure
