# Supplementary material for: Proteinuria Detected by Urine Dipstick Test as a Risk Factor for Atrial Fibrillation: A Nationwide Population-Based Study
Source: Sci Rep. 2017 Jul 24;7:6324. doi: 10.1038/s41598-017-06579-0 (PMC5524798; doi:10.1038/s41598-017-06579-0)

**Proteinuria Detected by Urine Dipstick Test as a Risk Factor for Atrial Fibrillation: A Nationwide Population-Based Study**

Woo-Hyun Lim, Eue-Keun Choi, Kyung-Do Han, Tae-Min Rhee, Hyun-Jung Lee, So-Ryoung Lee, Si-Hyuck Kang, Myung-Jin Cha, Seil Oh

***Supplemental Table.*** *International Classification of disease (ICD) codes used in the study for identifying patients’ baseline comorbidities*

| **Variables** | **ICD-10-CM codes** | **Definition** |
| --- | --- | --- |
| **Hypertension**^†^ | I10; I11; I12; I13; I14; I15 | One diagnosis during hospitalization or more than twice at outpatient clinics for the past year |
| **Diabetes mellitus (Type 2)** | E11-14 | One diagnosis and use of anti-diabetic medications fort the past year |
| **Dyslipidemia** | E78 | One diagnosis for the past year |
| **Ischemic heart disease** | I20-25 | One diagnosis during hospitalization or more than twice at outpatient clinics for the past year |
| **Myocardial infarction** | I21; I22 | One diagnosis during hospitalization or more than twice at outpatient clinics for the past three years |
| **Congestive heart failure** | I50 | One diagnosis for the past three years |
| **Stroke** | I63; I64 | One diagnosis during hospitalization or more than twice at outpatient clinics for the past year |
| **COPD** | J43; J44 | One diagnosis for the past three years |

COPD, chronic obstructive pulmonary disease; ICD-10-CM, International Classification of Diseases-Tenth Revision-Clinical Modification

**Supplementary Figure.** Schematic representation of enrolment and follow-up of study subjects


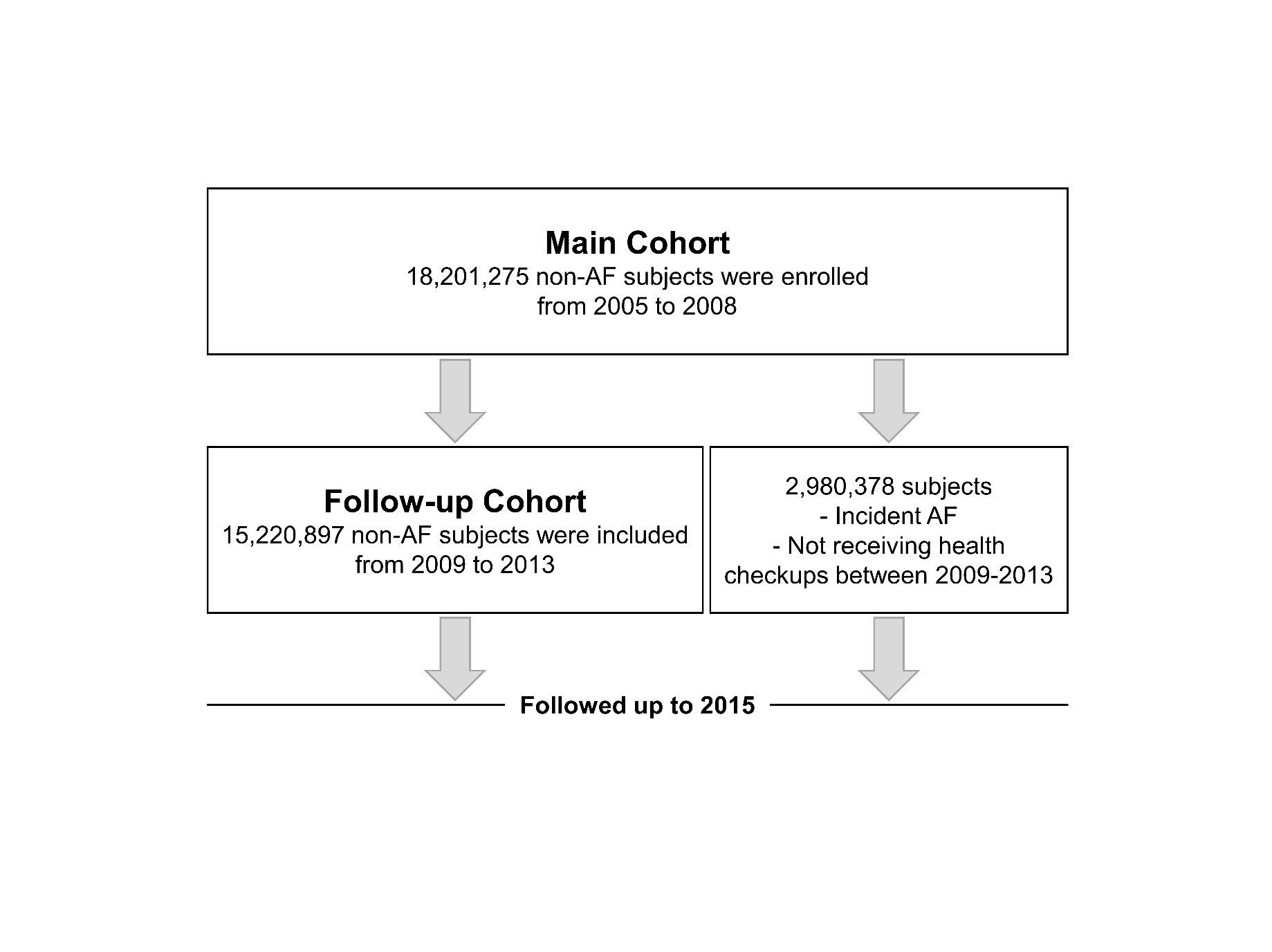

Supplement: Supplementary file 1 — Supplemental Table and Figure [file 41598_2017_6579_MOESM1_ESM.doc]
